# Ponderal index at birth and late life DNA methylation signature and its influence in cognition function: insights from the Age, Gene/Environment Susceptibility–Reykjavik Study

**DOI:** 10.1002/alz70860_098562

**Published:** 2025-12-23

**Authors:** Nigus G. Asefa, Tesfa Dejenie Habtewold, Valborg Gudmundsdottir, Vilmundur Gudnason, Fasil Tekola‐Ayele, Lenore J J. Launer

**Affiliations:** ^1^ Laboratory of Epidemiology and Population Sciences, National Institute on Aging, BALTIMORE, MD, USA; ^2^ Epidemiology Branch, Eunice Kennedy Shriver National Institute of Child Health and Human Development, National Institutes of Health, Bethesda, MD, USA; ^3^ University of Iceland, Reykjavik, Iceland; ^4^ Icelandic Heart Association, Kopavogur, Iceland; ^5^ National Institute of Child Health and Human Development, National Institutes of Health, Bethesda, MD, USA; ^6^ Laboratory of Epidemiology and Population Sciences, National Institute on Aging, Baltimore, MD, USA

## Abstract

**Background:**

A growing body of evidence suggests that an adverse prenatal environment is linked to poor health outcomes in later life. We performed birth‐size EWAS and developed a score and evaluated its relationship with cognitive function.

**Method:**

We analyzed data from 795 participants (mean age: 75.3 years; 58.4% female), a subset of the Age, Gene/Environment Susceptibility–Reykjavik Study (AGES‐RS) in Iceland, who had both birth size and late‐life DNAm data. Birth weight and length were collected from individuals born between 1914 and 1935. The PI was calculated as kg/m^3^. Whole blood‐derived DNA samples were collected between 2002 and 2006 and processed at the Huge‐F facility in the Netherlands. After quality control, DNAm data were available for 819,802 CpG sites, measured using the MethylEPIC V1 BeadChip. An epigenome‐wide association study (EWAS) was conducted, using the PI at birth as the exposure and late‐life DNA methylation levels as the outcome. The model was adjusted for age, sex, smoking status, white blood cell proportions, and batch effects. Additionally, we constructed an epigenetic score by weighting CpG methylation levels with EWAS effect sizes and explored its association with cognitive function.

**Result:**

No significant sex differences were found in mean PI (females: 25.8, males: 25.6 kg/m^3^). PI was significantly associated with methylation at CpG cg24617612 (*p* = 4.70e‐08). Additionally, PI was linked to 47 other CpG sites at a suggestive *p*‐value (*p* < 5e‐05). Hypergeometric testing revealed that these CpG sites were previously associated with various health conditions, including neurodevelopmental anomalies (*p* = 0.007), vascular inflammation (*p* = 0.01), adolescent victimization (*p* = 0.03), and organophosphate exposure (*p* = 0.03). Additionally, a one SD increase in the epigenetic score was associated with a 0.08‐point decrease (95% CI: 0.02‐0.014, *p* = 8.95e‐03) in the memory score.

**Conclusion:**

Late‐life DNA methylation patterns, potentially influenced by prenatal exposure to adverse conditions, were significantly associated with cognitive decline. These findings may highlight the potential role of early‐life factors in shaping epigenetic profiles and their implications for long‐term health outcomes.

